# Impact of the COVID-19 Pandemic on the Overall Health of Patients With Pre-diabetes and Diabetes

**DOI:** 10.7759/cureus.30477

**Published:** 2022-10-19

**Authors:** Goonja Patel, Sachi Singhal, Komaldeep Kaur, Pooja Jotwani, Ross Budziszewski, Courtney Fay

**Affiliations:** 1 Internal Medicine, Crozer-Chester Medical Center, Upland, USA; 2 Academic Affairs, Crozer-Chester Medical Center, Upland, USA

**Keywords:** obesity, hba1c, pandemic, covid-19, diabetic complications, diabetes

## Abstract

Coronavirus disease 2019 (COVID-19) impacted those with chronic diseases worldwide, especially those with diabetes. Very few studies have explored the effect of COVID-19 on diabetic patients’ health markers. The present retrospective study compared various health markers of diabetic patients before and during the COVID-19 pandemic. Patients (*N *= 511) displayed a significant increase in systolic blood pressure, hemoglobin A1c (HbA1c), diabetic medications, and dose of insulin (p* *< 0.05) as well as a decrease in low density lipoprotein (LDL) levels (p = 0.04). When patients were stratified by body mass index (BMI), those in higher BMI categories were more negatively impacted during the pandemic than those in lower categories. Results display the impact that COVID-19 had on the general well-being of diabetic patients, and should encourage providers to increase telehealth visits when in-person visits are not possible.

## Introduction

Early in the coronavirus disease 2019 (COVID-19) pandemic, it was determined that diabetic patients were at an increased risk of acquiring COVID-19 compared to the general population due to their compromised immune systems [1]. According to the national diabetes statistics report, diabetes affects 14.7% of the US adult population approximating around 34.1 million adults [2]. This number continues to grow as does the burden of diabetes-related complications. Diabetes affects multiple organ systems in the body and is associated with microalbuminuria, nephropathy, cardiovascular disease, peripheral vascular disease, neuropathy, retinopathy, and more [3]. Given that patients with diabetes often require medicine-driven health interventions, such as oral medications and insulin, regular physician visits are imperative to reduce microvascular and macrovascular comorbidities. 

While the negative impact of COVID-19 on those with chronic disease was apparent in early reports, many physicians and outpatient offices were closed during the peak of the pandemic, leading to a lapse in follow-up visits for patients with diabetes [4-5]. This posed a threat to the overall health of patients with diabetes as they require periodic monitoring to prevent the consequences of their disease process. The COVID-19 pandemic not only limited patient access to healthcare but also created barriers to behavior modification change. Notably, health centers, grocery stores, and gyms all either had limited or no access at the peak of the pandemic, often adding difficulty to maintaining healthy activity levels and diets [6-7]. Moreover, many patients suffered from loss of employment, increased levels of stress, and loss of access to proper monitoring of their comorbidities [8]. 

Few studies have examined the impact that COVID-19 had on diabetic patients’ health and compared specific health markers to those prior to the start of the pandemic [e.g., hemoglobin A1c, HbA1c, urine microalbumin, medications, BMI]. This study was conducted to assess the impact of the COVID-19 pandemic on the overall health of patients with diabetes. Specifically, the current study explores several health markers across the span of the pandemic to determine whether or not patients were negatively impacted by the COVID-19 pandemic. We hypothesized that the COVID-19 pandemic introduced a detrimental negative effect on patients with diabetes, which can result in an increase in diabetic complications longitudinally.

## Materials and methods

Data were collected using the electronic medical records of a large community outpatient internal medicine practice in the Northeast United States. Patients were gathered who matched the ICD codes for pre-diabetes and type 2 diabetes. The charts were reviewed to collect data. The data collected included age, sex, race, BMI, systolic blood pressure, diastolic blood pressure, HbA1c, urine microalbumin, LDL, number of diabetic medications, and dose of insulin. The sex was determined to be either male or female. The race category includes Caucasian/White, African-American/Black, Hispanic, American Indian/Alaska Native, Asian, and Unknown. The number of anti-diabetic medications included all oral and injectable antidiabetic agents. The dose of insulin included a total number of combined units for both long- and short-acting insulins. 

For each patient, data points from two different time periods were collected. Point A was prior to the start of the pandemic (January 2019-March 2020), and point B was a few months into the pandemic (June 2020-April 2021). If there were many data points for one variable during this time period, which was the case for patients who had more than one appointment during the selected time period, then an average was taken for the two most recent values in each time period. We excluded any patients with insufficient data for both time periods.

To compare both of the groups, an average value for each variable was collected using data from all of the patients for both time periods. A paired t-test was simulated to produce the study results that are interpreted below.

*​​*Data analysis**

Data were entered into Microsoft Excel (Seattle, Washington, USA). Patients who did not have sufficient data (i.e., data for both time periods) were excluded. Data from the two time periods were compared using paired t-tests to display any significant differences, and the size effect was measured using Cohen’s d. Then, patients were broken into BMI groups and within group changes were measured using paired t-tests. Statistical analyses were performed using SPSS Statistics Version 25 (IBM Corp., Armonk, NY).

## Results

Descriptive statistics

The current sample contained 511 patients, of which 45% (n = 230) were male and 55% (n = 281) were female. Patients on average were 58.31 years (standard deviation, SD = 12.83, range: 23-92 years). Race was determined as follows: White (n = 178), Black (n = 230), Hispanic (n = 9), American Indian/Alaskan Native (n = 6), Asian (n = 12), and unknown/not reported (n = 54).

Impact of COVID-19 on health markers

Paired t-tests revealed that the change in BMI, diastolic blood pressure, and urine microalbumin were not significantly different from Point A to Point B. Systolic blood pressure was significantly higher at Point B (M = 135.28) than Point A (M = 130.62): t(490) = -5.17, p < 0.001, d = 0.26. HbA1c levels also significantly increased from Point A to Point B: t(479) = -2.45, p = 0.015, d = 0.11. Impact on HbA1c was not calculated separately for patients with pre-diabetes versus patients with diabetes due to not having a significant amount of patients who fit into the pre-diabetic range. Additionally, LDL levels significantly decreased from Point A (95.99 mg/dL) to Point B (92.05 mg/dL): t(386) = 2.11, p = 0.036, d = 0.10. Lastly, levels of insulin and the number of diabetic medications both increased significantly (p < 0.05) from Point A to Point B. Table 1 shows the mean at both time points with associated p values.

**Table 1:** Health marker variables. HbA1c, hemoglobin A1c; LDL, low density lipoprotein

Variable	Point A	Point B	p-value
Body mass index	35.17(8.71)	34.95(8.61)	0.40
Systolic blood pressure	130.62(17.12)	135.28(18.16)	< 0.001
Diastolic blood pressure	78.90(10.83)	79.31(10.67)	0.45
HbA1c	7.87(2.15)	8.11(2.22)	0.015
Urine Microalbumin	256.98(2422.52)	162.67(528.82)	0.474
LDL	95.99(40.03)	92.05(40.68)	0.036
Diabetic medication	1.26(0.91)	1.60(0.95)	< 0.001
Insulin	16.88(24.74)	20.66(31.19)	0.018

Further classification based on obesity class 

The patient sample was also separated into groups based on BMI. The underweight class included a BMI <18.5 (n = 0); normal weight BMI was 18.5-24.9 (n = 36), overweight BMI was 25.0-29.9 (n = 106), class I obesity BMI was 30.0-34.9 (n = 133), class II obesity BMI was 35.0-39.9 (n = 94), and obesity class III BMI was >40.0 (n = 123). The groups were then compared using a paired test to evaluate changes from Point A to Point B.

Patients in the normal weight range (BMI = 18.5-24.9) reported no significant differences from the pre-post COVID pandemic. Patients in the overweight group (BMI = 25-29.9) reported minor, but statistically significant increase in diabetic medications used by 0.12 units: t(97) = -3.28, p < 0.001. In the obesity class I group (BMI = 30.0-34.9) there was a significant increase in systolic blood pressure by 5.58 mmHg (p = 0.001) and diabetic medications by 0.41 medications (p < 0.001). In the obesity class II group (BMI = 35.0-39.9) there was a significant increase in systolic blood pressure by 5.51 mmHg (p = 0.03) and diabetic medications by 0.35 medications (p < 0.001). Lastly, in the obesity class III group (BMI > 40.0) there was a significant increase in systolic blood pressure by 4.69 mmHg (p = 0.02), HbA1c by 0.5% (p = 0.01), and diabetic medications by 0.36 medications (p < 0.001). There was a significant decrease in BMI in this group by 0.89 points (p = 0.004).

## Discussion

In this retrospective study, we analyzed the overall health of patients with diabetes before and during the COVID-19 pandemic. There were no significant changes in BMI, diastolic blood pressure, or urine microalbumin when comparing data as a whole for all participants. However, results displayed statistically significant increases in systolic blood pressure, the number of diabetic medications being used, and the dose of insulin. There was also a statistically significant decrease in LDL levels. As obesity is a known cause of increased mortality and morbidity in patients with diabetes, we further separated our patients based on BMI class. Our results indicated that the patients with a BMI of 40 or more (Class III) showed significant worsening of HbA1c, diabetic medications, and systolic blood pressure.

According to the American Diabetes Association, the standard of care for diabetes should include a comprehensive assessment of complications, lifestyle management, glycemic control, medications, risk assessment, and prevention of diabetes-related complications [9]. The pandemic posed unique challenges for patients with diabetes and their ability to appropriately manage their disease. Many outpatient offices had to shut down during the peak of COVID-19 resulting in a large barrier to accessing care. A survey conducted by Zhao et al. which included 112 offices from 46 different countries reported that only 6.3% of offices kept their outpatient offices fully open throughout the pandemic [10]. Many gyms were also forced to close which eliminated the physical activity aspect of the behavioral modifications required to keep diabetes controlled [11]. In addition to these barriers, people refrained from leaving their houses in fear of contracting COVID-19 infection. This could explain the deterioration of health in patients with Class III obesity especially. This phenomenon was also demonstrated in an observational study conducted by Ruissen et al. which showed not only increased stress and anxiety but weight gain and loss of physical activity throughout the pandemic [11]. Another consequence of the pandemic was the loss of employment faced by individuals all over the country. As job shortages continued to grow, patients lost their income, health insurance, and the ability to pay for healthcare and medication-related expenses [12-13].

It is also important to mention that although there were significant deteriorations in many of the studied variables, this change was not as drastic as we originally expected. The negative aspect of the pandemic mentioned above did impact our patients with diabetes, but there were also key positive outcomes of this pandemic that we observed. One possible explanation is the increased popularity of telehealth medicine during the pandemic. There was a reported 154% increase in telehealth visits during March of 2020 compared to the year before [14]. As physician offices were closed for in-person visits, and many patients were afraid to come to the hospital due to the fear of being infected with COVID-19, physicians began to see more patients via telephone or video call. This allowed patients the comfort of speaking to a doctor from the safety of their own homes. All patients who previously had problems with transport or worked full-time jobs were able to address all their medication concerns from home. A patient satisfaction survey by Ramaswamy et al. concluded that the 8729% increase in video visits during the pandemic was associated with higher levels of patient satisfaction compared to the pre-pandemic period [15]. 

We hypothesize that another factor that led to better outcomes than predicted was that the COVID-19 pandemic inspired many to take better care of their health. We suspect that during a pandemic, many patients made more of an active effort to control their diabetes than perhaps they have done in the past due to fear of the consequences of being exposed to the COVID-19 virus as a patient with uncontrolled diabetes. 

This study carried many strengths, the most important being that it is one of the first studies to investigate the phenomenon of a pandemic affecting the health of patients with diabetes. The sample size used to collect data was satisfactory to observe the intended changes in our variables. We also had an even distribution in gender and obesity class which allowed our data to be evenly distributed. 

There are several ways this study could be improved further. Our study collected data from a single-center outpatient clinic. Collecting data from different centers with different locations and patient populations would provide a broadened view of the pandemic’s effect. In addition, when we collected data for the number of diabetic medications we did not differentiate between oral, injectable non-insulin, and insulin medications. A more detailed look into this could unveil further data points. Lastly, we did not have any group to serve as a control group. A possible improvement to this study could include comparing the group of patients with diabetes a year before the pandemic and seeing the change in their overall health even before the pandemic hit. This would allow us to isolate the effect of the pandemic even further.

## Conclusions

This study highlights the impact of a pandemic on the diabetic population, and the importance of close monitoring required in this population to avert morbidity and mortality. Diabetes has been a known risk factor for severe COVID-19 infection, but it was crucial to look at the effect of the pandemic itself on the overall health of these patients. Although initially, we thought that this project would only unveil negative outcomes of the pandemic on patients with diabetes, further investigations of the variables revealed that the COVID-19 pandemic instigated both positive and negative effects on their health. One thing is for certain, during periods of high stress, constant change, and poor conditions, it is imperative that patients with diabetes receive the same monitoring and access to treatment in order to avoid a decline in their health.
